# Independence and upper extremity functioning after spinal cord injury: a cross-sectional study

**DOI:** 10.1038/s41598-023-29986-y

**Published:** 2023-02-23

**Authors:** Lamprini Lili, Katharina S. Sunnerhagen, Tiina Rekand, Margit Alt Murphy

**Affiliations:** 1 Institute of Neuroscience and Physiology, Clinical Neuroscience, Rehabilitation Medicine, Sahlgrenska Academy, University of Gothenburg, Per Dubbsgatan 14, 3Rd Floor, 41345 Gothenburg, Sweden; 2 Department of Neurological Rehabilitation, Sahlgrenska University Hospital, Gothenburg, Sweden; 3 Department of Neurology, Haukeland University Hospital, Bergen, Norway; 4 Department of Occupational Therapy and Physiotherapy, Sahlgrenska University Hospital, Gothenburg, Sweden

**Keywords:** Neuroscience, Medical research, Neurology

## Abstract

Upper extremity functioning is important for achieving independence in activities of daily living (ADL). A better understanding of relationships between different aspects of independence in ADL after spinal cord injury (SCI) and upper extremity functioning is required to guide rehabilitation practices. To determine which aspects of independence in ADL are correlated with upper extremity functioning in individuals with cervical or thoracic SCI. A total of 25 adults (mean age 58.4 years, 72% men) with established cervical or thoracic SCI were recruited. Independence in ADL was assessed by Spinal Cord Independence Measure (SCIM-III) and upper extremity functioning by kinematic measures (movement time, smoothness, and wrist angle during drinking task), grip strength, Upper Extremity Motor and Sensory Score, Box and Block Test (BBT), Action Research Arm Test (ARAT), and Upper Extremity Basic Data Set (ISCI-Hand and ISCI-Shoulder). Spearman correlation coefficients were used for data analyses. The SCIM-self-care subscale, particularly the feeding and dressing items, correlated moderately (*r* ≥ 0.5) with movement time and smoothness, grip strength, ARAT, BBT, and ISCI-Hand. The SCIM-respiration/sphincter subscale and the SCIM-mobility showed very low and low correlations with upper extremity assessments. However, at item level, respiration and bed/wheelchair mobility showed moderate correlations. Independence in self-care as domain and feeding/dressing, respiration and bed/wheelchair mobility as separate items were dependent on upper extremity functioning in individuals with cervical or thoracic SCI. Movement time and smoothness along with BBT, grip strength, ARAT, and ISCI-Hand can be used as indicators of independence in ADL. These findings can provide guidance to clinical practice in selection of upper extremity assessments in the context for ADL in individuals with SCI.

## Introduction

Spinal cord injury (SCI) results not only in impairment of the extremities, but also in impairment of the trunk below the neurological level of injury. The management of SCI relies on an in-depth understanding of impairments, based fundamentally on the American Spinal Injury Association (ASIA) examination and classification^1–3^. However, although ASIA is the gold standard for clinical assessment, it has been criticized for problems with the psychometric properties of sensory function assessment of incomplete injuries, lack of sensitivity regarding functioning, and exclusion of certain segments (upper cervical, thoracic, and sacral), which in turn leads to gaps in the understanding of impairment in, for example, trunk motor function^4–6^.

In the later phases after SCI, both clinicians and patients tend to focus on activity limitations and independence in activities of daily living (ADL) rather than impairments^3,6^. According to the International Classification of Functioning, Disability and Health (ICF), the domain of activity can be divided into activity capacity (capacity to perform tasks and activities) and activity performance (actual use of the upper extremity in ADL)^7^. Assessment in different ICF domains is recommended^8^ because specific impairments (e.g., neurological injury level or grade of completeness) are not directly indicative of expected limitations in activity^3,6,9^.

To improve the clinical relevance of activity performance assessment, the Spinal Cord Independence Measure (SCIM)^10^ was developed, and the revised version of the SCIM (SCIM-III)^11,12^ is now recommended as a primary, valid, and reliable measure, specific for individuals with SCI^4,6^. For activity performance and independence in real life, the upper extremity plays a fundamental role^3,13^ and its restoration is ranked as one of the main priorities for individuals with cervical SCI^14,15^. Upper extremity activity performance assessed with the SCIM self-care subscale in individuals with cervical SCI has been shown to correlate with upper extremity functioning (i.e., including body functions-strength and activity capacity)^16^. The SCIM self-care subscale has also been used as an indirect indicator of upper extremity activity^17–22^. Activity performance assessed with the SCIM correlates with movement smoothness of reach-to-grasp^23^. Upper extremity activity capacity can be explained (between 59 and 81% of the variation) by kinematic measures of wrist angle, movement time, and smoothness^24^.

Although previous studies have examined some aspects of independence in ADL in respect with upper extremity functioning^16^, a more detailed understanding of the relationships between independence in activities at different levels and upper extremity functioning assessed with both objective kinematic measures and clinical assessments is still lacking^6,13^. This knowledge would guide clinicians in selection of appropriate upper extremity assessments when the goal is to measure outcomes related to independence in daily life.

Thus, the aim of this study was to determine which aspects of independence in ADL are correlated with upper extremity functioning in individuals with cervical or thoracic SCI.

## Methods

### Study design and participants

In this observational cross-sectional study, participants were recruited from an outpatient clinic at Sahlgrenska University Hospital in Gothenburg, Sweden, in 2018. Inclusion criteria were: cervical or thoracic SCI with grade of severity A, B, C, or D according to the ASIA Impairment Scale (AIS)^1,2^, injury present for more than 1 year, age ≥ 18 years, limited independence (SCIM-total score less than 100), and ability to use the upper extremity to some degree for everyday task such as drinking from a glass. Exclusion criteria were inability to communicate in Swedish and other psychological, neurological, or musculoskeletal comorbidities that could affect upper extremity use in ADL. The neurological level of the SCI was determined according to the International Standards for Neurological Classification of Spinal Cord Injury (ISNCSCI) developed by ASIA and the completeness of injury according to the ASIA Impairment Scale (AIS; A-E)^1,2^. This study was performed in accordance with the Declarations of Helsinki and approved by the Swedish Ethical Review Authority (registration number 408-17). All participants gave informed, written consent before recruitment in the study. The study was registered at researchweb.org (https://www.researchweb.org/is/vgr/project/260901) prior to participant enrollment. The reporting of this study conforms to the Strengthening the Reporting of Observational studies in Epidemiology (STROBE) statement^25^.

### Independence in ADL

Activity performance in the form of independence in ADL was assessed using SCIM-III^11,12^. SCIM-III includes 19 items divided into three subscales: (i) Self-care (SCIM-self-care), (ii) Respiration and Sphincter management (SCIM-respiration/sphincter) (iii) Mobility (SCIM-mobility). A score of 100 indicates independence in all SCIM-III areas. The SCIM-self-care, with a maximum score of 20, includes 6 items assessing feeding, upper and lower body bathing as well as dressing, and grooming. Both the SCIM-respiration/sphincter subscale (respiration, sphincter management-bladder and bowel, toileting) and the SCIM-mobility subscale (bed mobility; transfers and mobility indoors and outdoors) include 9 items with a maximum score of 40 points.

### Upper extremity functioning

The assessments of upper extremity covered different aspects of functioning according to the ICF framework^7^. The ASIA Upper Extremity Motor Score, the ASIA Upper Extremity Sensory Score, the grip strength as well as the kinematic variables of movement time (MT), number of movement units (NMU) also called smoothness, and wrist dorsiflexion angle covered the ICF domain of body functions. The Action Research Arm Test (ARAT), the International Spinal Cord Injury Upper Extremity Basic Data Set Form, and the Box and Block Test (BBT) covered the ICF domain of activity. For all assessments, data from the most affected upper extremity were included in the analysis. ARAT was used to determine the most affected arm, and when ARAT scores were equal in both arms, the pre-injury nondominant arm was considered the most affected arm.

### Clinical assessment of body functions

Grip strength was measured with a Jamar hydraulic hand dynamometer with analog readout in sitting position with elbow flexed 90 degrees and wrist between 0 and 30 dorsiflexion. The gravity of the dynamometer was supported by the tester or table. An average of three trials was calculated^26^. Grip strength has been shown to be associated with SCIM-total score^27^. According to the ASIA examination, strength in key muscles of the upper extremity was assessed with the manual muscle test with grades between 0 and 5 and summarized for each upper extremity in the Upper Extremity Motor Score with a maximum of 25 points^1,2^. Sensation in key points (according to the ASIA examination) in the upper extremity was rated on a scale ranging from 0 to 2 and summarized for each upper extremity in the Upper Extremity Sensory Score with a maximum of 32 points^1,2^. The grade of completeness was also assessed according to ASIA, and the data for this were derived from the medical records.

### Kinematic measures of body functions

**Figure 1:**
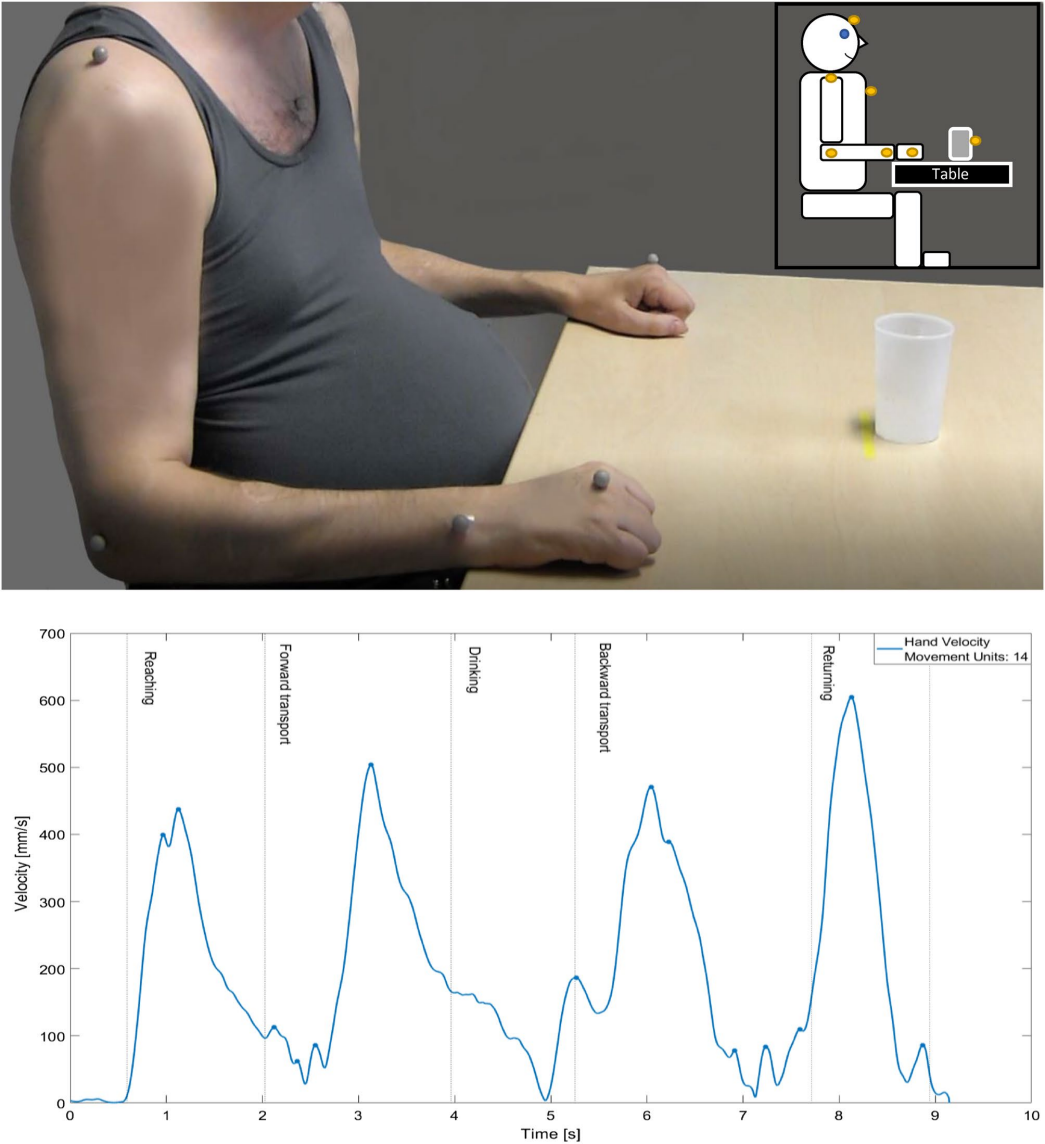
Initial position of the drinking task and placement of markers as well as tangential velocity profiles of hand markers and number of movement units in a participant with C6 AIS C spinal cord injury.

For the kinematic analysis of the standardized drinking task, a 5-camera optoelectronic motion capture system (Pro Reflex Motion Capture System, MCU240 Hz, Qualisys AB, Gothenburg, Sweden) was used^28,29^. The infrared light signals emitted by the cameras reflected by spherical markers placed at anatomical landmarks on the upper extremities (third metacarpophalangeal joint, styloid process of ulna, lateral epicondyle, upper middle part of acromion), forehead (notch between the eyebrows), upper part of sternum, and on a hard-plastic drinking glass (Fig. 1). The marker positions in 3D space were automatically identified and transferred for offline analysis using MATLAB (The Math Works Inc) software. The kinematic data were filtered using a 6 Hz second-order Butterworth filter in the forward and backward directions, resulting in a zero-phase distortion and fourth-order filtering before analysis. The drinking glass containing 100 ml of water was placed 30 cm from the edge of the table at the midline of the body (about 75% of the arm length). The standardized sitting position comprised approximately 90° knee, hip, and elbow flexion, the wrist in line with the edge of the table, and the back resting against the back of the chair. Trunk movement was not constrained and the participants using wheelchair were allowed to sit in their own chairs^30^ (Fig. 1). Participants were instructed to unimanually perform the task 8–10 times in a convenient speed as naturally as possible. For statistical analysis the mean of all performed trials were used^31^.

The drinking task comprised five phases: (i) reaching (including grasping), (ii) forward transport of the glass to the mouth, (iii) drinking, (iv) backward transport of the glass to the table, (v) and returning the hand to the starting position^28,29^. Movement time (MT) was measured for the entire drinking task including all movement phases^28,29^. The start and end of the movement was detected when the velocity of the hand marker exceeded or returned to 2% of the maximum velocity^28,29^. The number of movement units (NMU) was calculated by the tangential velocity profile of the hand marker for all transport phases except the drinking phase. A movement unit was defined as the difference between a local minimum and the next maximum velocity value that exceeded the amplitude limit of 20 mm/s, with a time between two consecutive peaks of at least 150 ms^28,29^. The NMU captures the repeated sub-accelerations and sub-decelerations during movement performance and can be defined as movement smoothness. The minimum NMU for the drinking task is 4 (one unit for each movement phase) and a higher NMU indicates a less smooth movement^30^. The maximum joint angle of dorsiflexion of the wrist was determined by the angle between the vectors connecting the hand and wrist as well as the elbow and wrist markers and was calculated for the reaching and forward transport phase^28,29^.

### Clinical assessments of activity capacity

Upper extremity activity capacity was assessed using the ARAT^32^. The ARAT includes 19 items hierarchically organised into 4 subscales (grasp, grip, pinch, and gross movement)^32^. The sum score ranges from 0 to 57, with higher scores indicating better performance^32^. The ARAT has been increasingly used in individuals with SCI^33,34^ and has showed moderate to good correlation with kinematic variables of movement time, smoothness, and maximum wrist angle^24^.

Gross manual dexterity was quantified using the BBT. The test score was the number of wooden blocks (2.5 cm) moved unimanually over a wooden divider from one compartment to another within one minute. The BBT is a reliable and simple assessment that measures the efficiency of movement regardless of the compensatory strategies used^35–38^.

The hand and shoulder classification scales from the International Spinal Cord Injury Upper Extremity Basic Data Set Form^39^, version 1.1^40^, developed by the International Spinal Cord Society (ISCoS), were used. The "Basic hand-upper extremity function" (ISCI-Hand) uses a 5-level scoring system based on the voluntary motor function of the upper extremity muscles required to perform common arm and hand movements such as reaching, grasping and manipulation^39,40^. The "Shoulder function classification" (ISCI-Shoulder) uses a 4-level scoring system based on observed shoulder and upper extremity function during arm positioning^39,40^. The ISCI Upper Extremity Basic Data Set Form has been tested and has shown excellent inter-rater reliability for evaluating cervical SCI^41^. ISCI-Hand has shown moderate correlation with kinematic measure of wrist angle^24^.

### Statistical analysis

Statistical analyses were performed using the IBM Statistical Package for Social Sciences™ (SPSS, version 24). Descriptive statistics were calculated for demographic and clinical characteristics. The two-sided significance level (alpha value) was set at p < 0.05. When both arms could perform the drinking task, data only from the more-affected arm (i.e., less total ARAT score) or the non-dominant arm was used. The preliminary analyses of included outcome measures showed non-normal distribution of all upper extremity assessments except for BBT. Therefore, Spearman correlation coefficient was used for correlation analysis between independence in ADL (i.e., SCIM-total, SCIM subscales, and each item) and upper extremity assessments (kinematic measures, clinical scales). The strength of correlation was interpreted as 0.00–0.25 (very low), 0.26–0.49 (low), 0.50–0.69 (moderate), 0.70–0.89 (high), and 0.90–1.00 (very high)^42^.

### Ethics approval and consent to participate

The study was approved by the Swedish Ethical Review Authority (registration number 408-17). We certify that all applicable institutional and governmental regulations concerning the ethical use of human volunteers were followed during the course of this research.

## Results

Of the 411 medical records screened, 216 individuals with SCI were eligible for the current study. Of those who responded to the telephone call (n = 134), 32 were interested in participating and 25 met the inclusion criteria and were included in the study^24^. The mean age of the study group was 58.4 years (range 44.6–72.2), 72% were men, and the mean time since injury was 17.5 (range 2.1–32.9) years. Of all participants, 80% had a traumatic injury and 68% had a cervical injury. The majority had motor complete injury (56%), although all grades of severity (injury level and completeness) were included except AIS E (Table 1). Four individuals with thoracic injuries and two individuals with cervical incomplete injury had full points in arm functioning according to the ARAT total score. Participants with incomplete AIS type D injuries all but one had a cervical SCI (Table 1).

**Table 1:** Background characteristics of the participants.

Characteristics	Participants (n = 25)
Age years, mean (SD)	58.4 (13.9)
Sex	
Male	18 (72%)
Female	7 (28%)
BMI, mean (SD)	25 (4.6)
Years since SCI, mean (SD)	17.5 (15.4)
Aetiology of lesion	
Traumatic	20 (80%)
Non-traumatic	5 (20%)
Level of SCI	
Cervical	17 (68%)
Thoracic	8 (32%)
Completeness of SCI	
AIS type A, B	14 (56%)
AIS type C, D	11 (44%)
Severity of SCI	
C1-C4 AIS type A, B, C	5 (20%)
C5-C8 AIS type A, B, C	5 (20%)
T1-T12 A, B, C	7 (28%)
AIS type D	8 (32%)
Hand surgery	8 (32%)
Impaired sensation (more affected hand)	18 (72%)
Impaired proprioception (more affected hand)	8 (33%)
Full points arm activity capacity (ARAT total score 57)	6 (24%)

*AIS type A, B, C, D* American Spinal Injury Association (ASIA) Impairment scale, *BMI* Body Mass Index, *SCI* Spinal Cord Injury.

According to the inclusion criteria, none of the participants was fully independent in ADL as assessed by the SCIM total score. Total SCIM scores ranged from 41.8 to 84.82 (mean 63.3, median 65). The scores of the SCIM subscales (self-care, respiration/sphincter, mobility) and the results of all upper extremity assessments are shown in Table 2.

**Table 2:** Independence in Activities of Daily Living (ADL) and upper extremity assessments.

Characteristics	Participants (n = 25)
Independence in ADL	
SCIM-self-care (0–20), median (Q1-Q3)	18 (10.5–18.5)
SCIM-respiration/sphincter (0–40), median (Q1-Q3)	27 (20–34)
SCIM-mobility (0–40), median (Q1-Q3)	18 (11–29.5)
SCIM-total (0–100), median (Q1-Q3)	65 (49.5–80.5)
Body Functions ICF domain-kinematic measures	
Number of Movement Units, mean (SD)	12.8 (12.7)
Movement Time, seconds, mean (SD)	8.3 (4.1)
Wrist angle, degree, mean (SD)	35.9 (15.5)
Body Functions ICF domain-clinical assessments	
Grip strength, kPa, mean (SD)	39.3 (34.6)
Upper Extremity Motor Score (0–25), median (Q1-Q3)	20 (15–25)
Upper Extremity Sensory Score (0–32), median (Q1-Q3)	25 (22–32)
Activity ICF domain-clinical assessments	
Action Research Arm Test (0–57), median (Q1-Q3)	52 (37.5–56.5)
Box and Block Test, mean (SD)	42.0 (17.3)
ISCI-Hand (1–5)	
No function (1)	0
Passive tenodesis (2)	2 (8%)
Active tenodesis (3)	3 (12%)
Active extrinsic (4)	4 (16%)
Active extrinsic-intrinsic (5)	16 (64%)
ISCI-Shoulder (A-D)	
No active placing or reaching	0
Severely limited but able to reach mouth/head	1 (4%)
Limited but able to reach mouth/head with difficulty	6 (24%)
Full range of movement and independent reaching	18 (72%)

*ICF* International Classification of Functioning, Disability and Health, *ISCI* International Spinal Cord Injury Upper Extremity Basic Data Set Form, *ISCI-Hand* basic Hand variable of the ISCI, *ISCI-Shoulder* Shoulder variable of the ISCI, *SCIM* Spinal Cord Independence Measure.

The Spearman correlation coefficients between the upper extremity assessments and the total SCIM score and the SCIM sub scores are reported in Table 3. In the ICF domain of body functions, kinematic measures of smoothness and movement time (*r* ≥ 0.6) as well as grip strength (*r* ≥ 0.5) showed moderate correlations with SCIM-self-care. In the ICF domain of activity, clinical assessments of ARAT, BBT, and ISCI-Hand showed moderate correlations (*r* ≥ 0.5). Correlations with SCIM-respiration/sphincter subscale were non-significant and low or very low. For the SCIM-mobility subscale, statistically significant but low correlations (*r* 0.3–0.5) were observed for grip strength and ISCI-Hand. SCIM-total showed low but statistically significant correlations with the kinematic measure of movement time and the ISCI-Hand classification score.

**Table 3:** Spearman coefficients for Spinal Cord Independence Measure (SCIM) subscale scores and upper extremity assessments.

Upper extremity assessments (n = 25)	SCIM-self-care	SCIM-respiration/sphincter	SCIM-mobility	SCIM-total
Body Functions ICF domain-kinematic measures				
Number of Movement Units	−** 0**.63**	− 0.20	− 0.34	− 0.34
Movement Time	− **0**.62**	− 0.12	− 0.48*	− 0.44*
Wrist angle	− 0.35	− 0.29	− 0.22	− 0.31
Body Functions ICF domain-clinical assessments				
Grip Strength	**0**.52**	0.13	0.40*	0.38
Upper Extremity Motor Score	0.40*	0.02	0.19	0.21
Upper Extremity Sensory Score	0.33	− 0.1	< 0.01	0.02
Activity ICF level-domain assessments				
Action Research Arm Test	**0**.56**	< − 0.01	0.33	0.29
Box and Block Test	**0**.55**	0.12	0.29	0.33
ISCI-Hand	**0**.52**	0.21	0.42*	0.42*
ISCI-Shoulder	0.37	− 0.23	− 0.22	− 0.16

**P < 0.01 *P < 0.05 Correlation coefficients > 0.5 are marked with bold.

*ICF* International Classification of Functioning, Disability and Health, *ISCI* International Spinal Cord Injury Upper Extremity Basic Data Set Form, *ISCI-Hand* basic Hand variable, *ISCI-Shoulder* Shoulder variable, *SCIM* Spinal Cord Independence Measure.

Respective Spearman correlation coefficients between the upper extremity assessments and the total SCIM score and the SCIM sub scores only in participants with cervical SCI (n = 17) are reported in the Supplementary Table 1. A separate statistical analysis with thoracic SCI alone lacked statistical power due to the small (n = 8) sample size.

Table 4 shows the correlation coefficients between the upper extremity assessments and each item of the SCIM-self-care subscale. Moderate and high correlations were observed for feeding and for the upper and lower body dressing items with the kinematic measures of movement time and smoothness, grip strength, ARAT, and BBT. The Upper Extremity Motor Score and the ISCI-Hand showed moderate correlations with the feeding and upper body dressing items. The kinematic measure of wrist angle, the Upper Extremity Sensory Score, and the ISCI-Shoulder showed moderate correlations with dressing of the upper body alone. The upper body dressing correlated with all upper extremity functioning assessments. The SCIM self-care items of bathing and grooming showed statistically significant but low correlations with some upper extremity assessments.

**Table 4:** Spearman correlation coefficients for each item of the SCIM-self-care subscale and the upper extremity assessments.

Upper extremity assessments (n = 25)	Feeding	Bathing upper	Bathing lower	Dressing upper	Dressing lower	Grooming
Body Functions ICF level-kinematic measures						
Number of Movement Units	−** 0**.62**	− 0.35	− 0.46*	− **0**.76**	− 0.70**	− 0.34
Movement Time	−** 0**.62**	− 0.39	− 0.44*	− **0**.69**	− **0**.61**	− 0.27
Wrist angle	− 0.48*	− 0.31	− 0.24	− 0.51**	− 0.36	− 0.20
Body Functions ICF level-clinical assessments						
Grip strength	**0**.68**	0.40*	0.44*	**0**.54**	**0**.54**	0.35
Upper Extremity Motor Score	**0**.53**	0.21	0.27	**0**.57**	0.45*	0.29
Upper Extremity Sensory Score	0.35	0.06	0.08	**0**.56**	0.37	0.38
Activity ICF level-clinical assessments						
Action Research Arm Test	**0**.70**	0.33	0.35	**0**.73**	**0**.53**	0.38
Box and Block Test	**0**.59**	0.32	0.32	**0**.74**	**0**.59**	0.35
ISCI-Hand	**0**.61**	0.38	0.39	**0**.57**	0.50*	0.45*
ISCI-Shoulder	0.30	− 0.10	0.11	**0**.69**	0.39	0.27

**P < 0.01 *P < 0.05 Correlation coefficients > 0.5 are marked with bold.

*ICF* International Classification of Functioning, Disability and Health, *ISCI* International Spinal Cord Injury Upper Extremity Basic Data Set Form, *ISCI-Hand* basic Hand variable, *ISCI-Shoulder* Shoulder variable, *SCIM* Spinal Cord Independence Measure.

Table 5 shows the correlation coefficients between the upper extremity assessments and each item of the SCIM-respiration/sphincter subscale. The kinematic measures of movement time and smoothness as well as BBT showed moderate correlations with the respiration item. Movement time was also moderately correlated with toilet use item. The bladder and bowel items had non-significantly low or very low correlations with upper extremity functioning.

**Table 5:** Spearman correlation coefficients for each item of the SCIM-respiration/sphincter subscale and the upper extremity assessments.

Upper extremity assessments (n = 25)	Respiration	Bladder	Bowel	Toilet use
Body Functions ICF level-kinematic measures				
Number of Movement Units	− 0.50*	− 0.06	0.24	− 0.48*
Movement Time	− 0.53**	− 0.15	0.18	− 0.66**
Wrist angle	− 0.50*	− 0.21	− 0.16	− 0.30
Body Functions ICF level-clinical assessments				
Grip strength	0.36	0.18	− 0.14	0.34
Upper Extremity Motor Score	0.47*	< − 0.01	− 0.10	0.19
Upper Extremity Sensory Score	0.45*	− 0.09	− 0.23	− 0.06
Activity ICF level-clinical assessments				
Action Research Arm Test	0.47*	0.10	− 0.29	0.39
Box and Block Test	0.56**	0.08	− 0.11	0.46*
ISCI-Hand	0.44*	0.32	− 0.04	0.29
ISCI-Shoulder	0.43*	− 0.31	− 0.30	0.09

**P < 0.01 *P < 0.05 Correlation coefficients > 0.5 are marked with bold.

*ICF* International Classification of Functioning, Disability and Health, *ISCI* International Spinal Cord Injury Upper Extremity Basic Data Set Form, *ISCI-Hand* basic Hand variable, *ISCI-Shoulder* Shoulder variable.

As for the SCIM-mobility subscale, the bed mobility and wheelchair mobility items showed moderate correlations with the kinematic measure of movement time (Table 6). Moderate correlations were also found between smoothness of movement, grip strength, ARAT, BBT, ISCI-Hand, and the bed mobility item. ISCI-Hand also correlated moderately with ground wheelchair mobility.

**Table 6:** Spearman correlation coefficients for each item of the SCIM-mobility subscale and the upper extremity assessments.

Upper extremity assessments (n = 25)	Bed mobility	Bed-wheel chair	Toilet-wheel chair	In doors mobility	Moderate distances	Out doors mobility	Stairs	Car-wheel chair	Ground-wheel chair
Body Functions ICF level-kinematic measures									
Number of Movement Units	− 0.59**	− 0.45*	− 0.48*	− 0.17	− 0.15	− 0.23	− 0.17	− 0.35	− 0.45*
Movement Time	− 0.61**	− 0.58**	− 0.58**	− 0.31	− 0.29	− 0.34	− 0.30	− 0.51**	− 0.58**
Wrist angle	− 0.26	− 0.34	− 0.36	− 0.13	− 0.12	0.04	− 0.18	− 0.22	0.18
Body Functions ICF level-clinical assessments									
Grip strength	0.61**	0.43*	0.35	0.17	0.17	0.32	0.19	0.39	0.42*
Upper Extremity Motor Score	0.37	0.25	0.25	0.06	0.05	0.05	0.12	0.17	0.31
Upper Extremity Sensory Score	0.16	0.13	0.14	− 0.11	− 0.09	− 0.08	− 0.07	0.03	− 0.02
Body Functions ICF level-clinical assessments									
Action Research Arm Test	0.57**	0.45*	0.37	0.25	0.12	0.19	0.15	0.37	0.42*
Box and Block Test	0.52**	0.44*	0.49*	0.12	0.06	0.12	0.14	0.34	0.38
ISCI-Hand	0.51**	0.45*	0.43*	0.21	0.24	0.37	0.28	0.40*	0.50*
ISCI-Shoulder	0.25	− 0.01	< − 0.01	− 0.32	− 0.29	− 0.26	− 0.44*	− 0.17	0.01

**P < 0.01 *P < 0.05 Correlation coefficients > 0.5 are marked with bold.

*ICF* International Classification of Functioning, Disability and Health, *ISCI* International Spinal Cord Injury Upper Extremity Basic Data Set Form, *ISCI-Hand* basic Hand variable, *ISCI-Shoulder* Shoulder variable, *SCIM* Spinal Cord Independence Measure.

## Discussion

The results of this cross-sectional study showed that independence in activities of daily living and, especially, independence in self-care correlated with upper extremity functioning as measured in the ICF domain of body functions and activity. The SCIM-self-care subscale and, especially the feeding and dressing items correlated with kinematic measures of movement time and smoothness, grip strength, ARAT, BBT, and ISCI-Hand. Correlations with the SCIM-respiration/sphincter subscale were low and not statistically significant. However, at the item level, moderate correlations were observed for movement time, smoothness, and BBT with respiration item and for movement time with toilet use item. The SCIM-mobility subscale showed low but statistically significant correlations with grip strength and ISCI-Hand. At the item level, moderate correlations were observed for the items bed mobility and wheelchair transfers with movement time, smoothness, grip strength, ARAT, BBT, and ISCI-Hand.

Our results showed that independence in self-care, rather than independence in mobility and in respiration and sphincter management, correlated with upper extremity functioning in individuals with cervical or thoracic SCI. This finding is in line with previous research that mainly included individuals with tetraplegia, even though the strength of correlations varies between different study populations^16,20,21,43^. One possible explanation for these differences could be the degree of independence in ADL in each specific study population. For example, correlations between upper extremity functioning and the SCIM-self-care subscale were stronger in studies that included only participants with tetraplegia, in which the participants’ degree of independence in ADL was lower^16,44^, compared with studies such as ours that also included participants with paraplegia^20^, in which the participants’ degree of independence was higher.

In the current study, the SCIM-self-care items of feeding and dressing showed the strongest correlations with upper extremity functioning. Similar results were also reported by Rudhe et al.^16^, who found that the upper body dressing and grooming items correlated with motor score and hand capacity tests^16^. In addition to the self-care items, the bed and wheelchair mobility items also showed moderate correlations with upper extremity functioning in the current study. This finding is consistent with a study in individuals with cervical complete or incomplete SCI after hand surgery, in which the feeding and wheelchair mobility items were selected as indicators of functional independence^45^. On the SCIM-respiration/sphincter subscale, at the item level, our study showed that the independence in respiration item correlated significantly with several upper extremity assessments. This interesting finding is clinically relevant considering the role of muscles stabilizing the shoulder girdle, such as the latissimus dorsi and serratus muscles, in respiration.

In the current study, kinematic measures were included to objectively characterize and quantify upper extremity movements during a purposeful task. Among the upper extremity assessments, measures of movement time and smoothness showed the strongest correlations with the SCIM-self-care subscale. In addition, at the item level, independence in feeding, dressing, wheelchair/bed mobility, respiration and toilet use correlated with movement time and smoothness. These results are novel and show that independence in these activities is closely related to the smoothness of arm movements and the time required to perform a standardized everyday task, such as drinking from a glass. While movement time is easily captured in clinical settings, movement smoothness is a measure that requires more advanced camera-based analysis. Previous research has shown strong correlation between movement time and smoothness^24^. This suggests that standardized, timed everyday tasks, such as the drinking task, could probably be used as a potential indicator of independence in ADL.

Grip strength is another simple clinical test that showed moderate correlation with independence in self-care and at the item level with independence in feeding, dressing, and mobility in bed. Grip strength has been previously reported to correlate with SCIM-total score^27^. In the ICF domain of activity, the clinical assessments BBT, ARAT, and ISCI-Hand correlated moderately with independence in self-care and at the item level with independence in feeding, dressing, mobility in bed, and respiration. Given these results, all clinical assessments that showed moderate correlations with independence in ADL can be recommended as clinical proxies for independence in ADL. Considering that all participants in this study were able to move at least one block on the BBT, while four participants scored 0 on the grip strength test, and that the ARAT requires a longer administration time, the BBT could be recommended as a quick and easy clinical assessment after SCI, especially if the focus is on independence in ADL and not on movement quality or strategy used^36,46^.

### Strengths and limitations

In this study, the ICF framework was used to guide the selection of upper extremity assessments, which strengthens the study by providing clinicians with a more nuanced picture of the correlations across different functioning domains and measures. Most of the assessments used in the current study are well established, which increases the clinical validity of the results. Our approach of examining independence at the item level can also be considered a strength, as it provides further insight into the different domains and categories of independence in ADL. When item scores are summed in the SCIM total score or subscale scores, some information is lost^16^. In addition, our study is strengthened by kinematic analysis during a daily task, which adds a novel perspective to the results in relation to independence^30^.

The results in this study can only be generalized to a population of individuals with complete or incomplete SCI at the cervical or thoracic level with a relatively high degree of independence (mean SCIM score of 63 out of 100). The study sample was heterogeneous and included both cervical and thoracic injuries with varying completeness and severity of injury, which may be considered a limitation when the focus is on impairment. However, the focus of the current study was on independence in ADL and upper extremity functioning after SCI. Therefore, dependence in ADL rather than neurological level was selected as one of the inclusion criteria. Based on these criteria, 8 participants with thoracic injuries were also included in the study because they had a lower SCIM total score than 100. Of these 8 participants with decreased SCIM due to thoracic injuries, 4 had limitations in upper extremity functioning. This is not surprising given the importance of trunk stabilization to upper extremity motor control^47^. Future studies with larger samples are needed to determine the extent to which upper extremity functioning may be limited in individuals with thoracic SCI.

## Conclusions

Independence in self-care including feeding, dressing as well as respiration, and mobility in bed and wheelchair correlates with upper extremity functioning in individuals with cervical or thoracic SCI. In clinical settings, grip strength, BBT, ARAT, and ISCI-Hand as well as timed tests of functional tasks, such as drinking, can be used as indicators of independence in ADL. This new, deeper understanding of upper extremity functioning in the context of independence can guide the selection of assessments and rehabilitation interventions in clinical practice.

## Supplementary Information

Supplementary Information.

## Data Availability

The data that support the findings of this study are available via corresponding author on a reasonable request.

## Abbreviations

ADL: Activities of Daily Living
ASIA: American Spinal Injury Association
AIS: ASIA Impairment scale
ARAT: Action Research Arm Test
BBT: Box and Block Test
BMI: Body Mass Index
ICF: International Classification of Functioning, Disability and Health
ISCI: International Spinal Cord Injury Upper Extremity Data Set
ISCI-Hand: Basic Hand variable of the International Spinal Cord Injury Upper Extremity Data Set
ISCI-Shoulder: Shoulder variable of the International Spinal Cord Injury Upper Extremity Data Set
ISCoS: International Spinal Cord Society
ISNCSCI: International Standards for Neurological Classification of Spinal Cord Injury
NMU: Number of Movement Units
MT: Movement Time
SCI: Spinal Cord Injury
SCIM-III: Spinal Cord Independence Measure version III
SCIM-self-care: Spinal Cord Independence Measure self-care subscale
SCIM-respiration/sphincter: Spinal Cord Independence Measure respiration and sphincter management subscale
SCIM-mobility: Spinal Cord Independence Measure mobility subscale
SCIM-total: Spinal Cord Independence Measure total score
STROBE: Strengthening the Reporting of Observational studies in Epidemiology
UEMS: Upper Extremity Motor Score
UESS: Upper Extremity Sensory Score
