# Supplementary material for: Independence and upper extremity functioning after spinal cord injury: a cross-sectional study
Source: Sci Rep. 2023 Feb 23;13:3148. doi: 10.1038/s41598-023-29986-y (PMC9950049; doi:10.1038/s41598-023-29986-y)
Supplement: Supplementary file 1 — Supplementary Information. [file 41598_2023_29986_MOESM1_ESM.docx]

APPENDIX

**Supplementary Table 1.** Spearman coefficients for Spinal Cord Independence Measure (SCIM) subscale scores and upper extremity assessments.

| **Upper extremity assessments**  **(n=17 with cervical SCI)** | **SCIM-**  **self-care** | **SCIM-**  **respiration/sphincter** | **SCIM-**  **mobility** | **SCIM-**  **total** |
| --- | --- | --- | --- | --- |
| **Body Functions ICF domain – kinematic measures** | | | | |
| Number of Movement Units | **-0.69^**^** | -0.33 | **-0.57^*^** | **-0.55^*^** |
| Movement Time | **-0.65^**^** | -0.20 | **-0.48^*^** | **-0.43^*^** |
| Wrist angle | **-0.59^*^** | **-0.58^*^** | -**0.60**^*^ | **-0.63^*^** |
| **Body Functions ICF domain – clinical assessments** | | | | |
| Grip Strength | **0.51**^*^ | 0.45 | **0.71^**^** | **0.61^*^** |
| Upper Extremity Motor Score | 0.43 | 0.33 | **0.59^*^** | **0.48^*^** |
| Upper Extremity Sensory Score | 0.37 | 0.17 | 0.26 | 0.22 |
| **Activity ICF level – domain assessments** | | | | |
| Action Research Arm Test | **0.64^**^** | 0.28 | **0.59^*^** | **0.50^*^** |
| Box and Block Test | **0.67^**^** | 0.33 | 0.34 | 0.45 |
| ISCI-Hand | **0.58^*^** | 0.47 | **0.68^**^** | **0.60^*^** |
| ISCI-Shoulder | 0.34 | -0.18 | -0.26 | -0.14 |

***P*<0.01 **P*<0.05 Correlation coefficients > 0.5 are marked with bold.

*ICF* International Classification of Functioning, Disability and Health, *ISCI* International Spinal Cord Injury Upper Extremity Basic Data Set Form, *ISCI-Hand* basic Hand variable, *ISCI-Shoulder* Shoulder variable, *SCIM* Spinal Cord Independence Measure.
